# Microbial hydrogen cycling in agricultural systems – plant beneficial or detrimental?

**DOI:** 10.1111/1751-7915.14300

**Published:** 2023-06-24

**Authors:** Zahra F. Islam, Chris Greening, Hang‐Wei Hu

**Affiliations:** ^1^ School of Agriculture, Food and Ecosystem Sciences, Faculty of Science The University of Melbourne Parkville Victoria Australia; ^2^ ARC Research Hub for Smart Fertilisers The University of Melbourne Parkville Victoria Australia; ^3^ Department of Microbiology Biomedicine Discovery Institute, Monash University Clayton Victoria Australia; ^4^ Centre to Impact AMR Monash University Clayton Victoria Australia; ^5^ SAEF: Securing Antarctica's Environmental Future Monash University Clayton Victoria Australia

## Abstract

Hydrogen‐oxidising bacteria play a key role in maintaining the composition of gases within the atmosphere and are ubiquitous in agricultural soils. While studies have shown that hydrogen accumulates in soil surrounding legume nodules and the soil surface, soils as a whole act as a net sink for hydrogen, raising questions about how hydrogen is internally recycled by soils. Can the energy derived from hydrogen oxidation be directly funnelled into plants to promote their growth or does it only act as a booster for other plant‐growth promoting bacteria? Moreover, while the fertilisation effect of hydrogen on plants has previously been shown to be beneficial, questions remain about the upper limit of hydrogen uptake by plants before it becomes detrimental. Agricultural practices such as fertilisation may impact the balance of hydrogen‐oxidisers and hydrogen‐producers in these ecosystems, potentially having detrimental effects on not only agricultural land but also global biogeochemical cycles. In this perspectives piece, we highlight the importance of understanding the contribution of hydrogen to agricultural soils and the effects of agricultural practices on the ability for bacteria to cycle hydrogen in agricultural soils. We propose a framework to gain better insights into microbial hydrogen cycling within agroecosystems, which could contribute to the development of new agricultural biotechnologies.

## Graphical abstract

Hydrogen‐oxidising bacteria play a key role in maintaining the composition of gases within the atmosphere and are ubiquitous in agricultural soils. Agricultural practices such as fertilisation may impact the balance of hydrogen‐oxidisers and hydrogen‐producers in these ecosystems, potentially having detrimental effects on not only agricultural land but also global biogeochemical cycles. In this perspectives piece, we highlight the importance of understanding the contribution of hydrogen to agricultural soils and the effects of agricultural practices on the ability for bacteria to cycle hydrogen in agricultural soils.

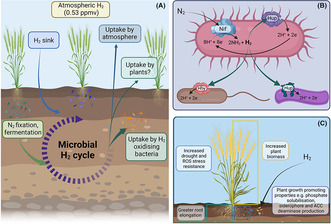

## THE IMPORTANCE OF HYDROGEN CYCLING WITHIN AGRICULTURAL SYSTEMS

**FIGURE 1:**
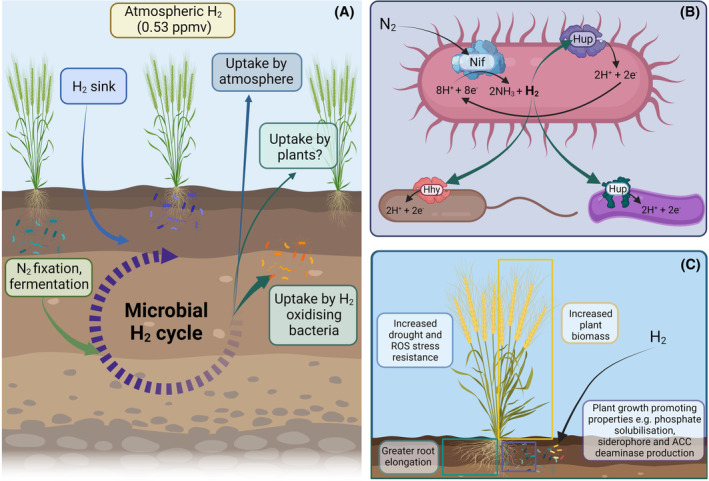
Overview of microbial hydrogen cycling within agricultural soils and predicted pathways of hydrogen removal from soil. (A) The concentration of hydrogen is maintained at approximately 0.53 parts per million by volume (ppmv) through various hydrogen production and removal processes in the soil. Hydrogen input into the soil microbial hydrogen cycle includes via hydrogen deposition from the atmosphere (indicated by the light blue arrow), nitrogen (N_2_) fixation and fermentation reactions (light green arrow). The produced hydrogen can then be taken up by hydrogen oxidising bacteria or by plants (dark green arrows) or it can be emitted into the atmosphere (dark blue arrow). (B) Proposed mechanisms of bacteria‐mediated soil hydrogen cycling. Hydrogen is produced as a by‐product of nitrogen fixation by nitrogenase enzymes (Nif). The excess hydrogen can then potentially be removed either through an internal hydrogen uptake hydrogenase (Hup) or by neighbouring bacteria that contain either a high affinity (e.g. Hhy) or low affinity (e.g. Hup) hydrogenase. (C) Proposed mechanisms by which hydrogen fertilisation occurs in crops. The direct effects of molecular hydrogen on crops include greater root elongation, increased plant biomass and improved resistance to drought and reactive oxygen species (ROS)‐mediated stress. Indirect effects include downstream effects of hydrogen oxidation on plant growth promoting rhizobacteria including greater phosphate solubilisation, siderophore and 1–aminocyclopropane–1–carboxylate (ACC) deaminase production. Figure created using BioRender.

Diverse bacteria within the soil, including those associated with plants, have been shown to use hydrogen (H_2_) as an energy source to support their growth and survival (Greening et al., 2022). While numerous studies have explored how soil‐borne organisms use hydrogen for their own energy requirements in a range of terrestrial ecosystems (Fan et al., 2022; Greening et al., 2022; Islam et al., 2020), less is known about how plant‐associated bacteria that cycle hydrogen affect agricultural productivity. Although soil ecosystems contribute a small proportion of atmospheric H_2_ emissions (~2–5 Tg/year), they are a net sink for atmospheric H_2_, accounting for approximately 75% of hydrogen uptake (~60 Tg per year) (Greening et al., 2022; Wang et al., 2020). The main contributor to soil H_2_ emissions are agricultural soils, which can contribute between 0.9 and 1.2 Tg of H_2_ per year to the atmosphere, due in part to the production of H_2_ as a by‐product of biological nitrogen fixation in the rhizosphere (Xu et al., 2021). With only a small amount of hydrogen produced by soils being released to the atmosphere (Figure 1A) (Wang et al., 2020), it is likely that closely associated rhizosphere or endophytic bacteria use the excess hydrogen produced by nitrogen (N_2_) fixing rhizobia that lack hydrogenases (Figure 1B). As it is expected that some of these bacteria act as plant‐growth promoters (PGP), understanding how hydrogen oxidation can fuel their abilities to promote plant growth is of great importance.

Fertilisation by endogenously produced H_2_ benefits plant growth, particularly in regions with high inorganic fertiliser usage or intensive legume cropping. Numerous experimental reports have demonstrated this fertilisation effect, with the main benefits including increased plant biomass, greater root elongation and increased stress resistance. This fertilisation also results in the proliferation of plant‐growth promoting rhizobacteria (PGPR) that can solubilise phosphorus, produce 1‐aminocyclopropane‐1‐carboxylate (ACC) deaminase, enhance siderophores, and have antifungal activity (Figure 1C) (Fan et al., 2022; Li et al., 2018; Maimaiti et al., 2007). It is thought that H_2_ may directly influence plant physiology through (i) modulation of the nitric oxide (NO) or auxin signalling pathways leading to favourable root elongation and development; (ii) modulation of enzymes involved in ameliorating reactive oxygen species‐related (ROS) and drought stress, such as those involved in stomatal closure; and (iii) crosstalk with other signalling molecules such as carbon monoxide (CO), which are involved in plant development (Li et al., 2018). While direct effects of H_2_ on plants may contribute to enhanced stress resistance, stronger effects are likely to occur through modulation of rhizosphere and potentially phyllosphere microbial communities. This reflects that H_2_ supporting the growth and survival of both PGPR and non‐PGPR microbial community members is likely to be the major driver for rhizosphere‐mediated growth enhancement (Fan et al., 2022). Although currently there is no evidence that excess hydrogen can cause negative effects on plant physiology and rhizosphere composition, it is important to consider the optimal H_2_ dosing concentrations when designing future agricultural biotechnologies so as to not contribute to the tropospheric burden of excess H_2_ or potentially cause the proliferation of H_2_ oxidising plant pathogens. The wide‐reaching benefits of H_2_‐based fertilisation previously demonstrated in various crops highlight the importance of understanding not only how plants respond to excess H_2_, but also how the balance of hydrogen‐oxidising and hydrogen‐producing members within the plant–soil microbiome might be affected.

## THE MOLECULAR BASIS OF AGRICULTURAL MICROBIAL HYDROGEN CYCLING

Research into rhizosphere and phyllosphere bacteria has revealed that numerous taxa harbour H_2_ oxidation genes, including those associated with agriculturally relevant crops (Kanno et al., 2016; Maimaiti et al., 2007). The most prevalent [NiFe]‐hydrogenase subtypes associated with plant‐associated bacteria and the surrounding soil are the low‐affinity Group 1d (also known as Hup) that recycle endogenously‐produced H_2_, the high‐affinity Group 1 h (also known as Hhy) that consume atmospheric H_2,_ and to a lesser extent, the high‐affinity Group 2a hydrogenases (also known as Huc) that can consume both atmospheric and elevated H_2_ (Fan et al., 2022; Greening et al., 2022; Kanno et al., 2016). Some rhizobia, known as Hup^+^ strains, internally recycle the majority of H_2_ produced as a by‐product of the nitrogenase reaction (Maimaiti et al., 2007). However, given most N_2_‐fixing rhizobia lack Hup, it is thought that the H_2_ produced through their nitrogenase reaction is consumed by surrounding rhizosphere bacteria, potentially resulting in a natural fertilisation effect. In particular, the consumption of nitrogenase‐derived H_2_ is likely reliant on bacteria that possess both Hup and Hhy hydrogenases to consume both excess and atmospheric concentrations of H_2_, respectively (Greening et al., 2022; Maimaiti et al., 2007).

A range of rhizosphere‐associated bacteria have been shown to grow on elevated concentrations of H_2_. Maimaiti et al. (2007) comprehensively characterised 18 H_2_‐oxidising bacterial isolates and their plant‐growth promotion capacity, including their ability to increase root elongation in wheat seedlings, as well as increase leaf number and plant biomass in *Arabidopsis thaliana* (Maimaiti et al., 2007). Although they did not specifically identify the type of hydrogenase present in their isolates, which belonged to the genera *Variovorax*, *Flavobacterium* and *Burkholderia*, genomic surveys have determined that their hydrogen oxidation capabilities are likely due to the low‐affinity Hup hydrogenase (Greening et al., 2016; Maimaiti et al., 2007). By coupling hydrogen oxidation to carbon fixation in species containing both enzymes, the existing evidence suggests that hydrogenotrophic chemosynthesis is a viable energy generation strategy for bacteria within agricultural soils (Greening et al., 2022; Maimaiti et al., 2007).

It is also increasingly appreciated that microbial uptake of atmospheric H_2_ can benefit the rhizosphere. To date, the capacity of plant‐associated bacteria to oxidise atmospheric concentrations of hydrogen in soil microcosms has been extensively studied by Kanno et al. (2016). The authors demonstrated that seven *Streptomyces* isolates from *Capsella bursa‐pastoris*, *Arabidopsis thaliana* and *Oryza sativa*, could oxidise atmospheric concentrations of hydrogen using Hhy (Kanno et al., 2016). It should be noted that the *Streptomyces* genus has only been shown to oxidise atmospheric H_2_ after sporulation, suggesting that Hhy supports streptomycete persistence in plant tissue and soils (Greening et al., 2022; Kanno et al., 2016). While Kanno et al. demonstrated high‐affinity hydrogen oxidation in plant tissue (Kanno et al., 2016), the majority of hydrogen oxidised was within the soil portion of the microcosm, highlighting the significance of soil‐mediated hydrogen oxidation (Greening et al., 2022; Kanno et al., 2016). Interestingly, Kanno et al. did not isolate or validate the role of bacteria capable of oxidising atmospheric concentrations of hydrogen using other types of [NiFe]‐hydrogenases. Comparatively, the role of Huc, which is capable of both atmospheric and elevated H_2_ oxidation, within agricultural soils is less defined, and likely to contribute less than Hup and Hhy to overall H_2_ concentrations within the soil. Nevertheless, in addition to soil‐borne bacteria, Huc has also been identified in a small number of plant‐associated rhizobacteria, including lineages capable of symbiotic and free‐living N_2_ fixation such as *Mesorhizobium*, *Bradyrhizobium* and *Frankia* (Islam et al., 2020). The metabolic benefit of H_2_ oxidation to bacteria likely leads to a competitive advantage, which could be problematic if H_2_‐oxidising species are also plant‐pathogens, such as *Streptomyces scabiei*, which has been shown via genomic surveys to encode Hhy (Greening et al., 2016). Thus, future studies investigating the capacity of plant‐associated bacteria to use H_2_ must consider both bacteria capable of hydrogenotrophic growth on elevated H_2_, such as directly surrounding legumes, as well as those that persist on atmospheric levels of the gas.

Several studies have investigated the effects of H_2_ oxidation on the composition and function of soil and rhizosphere communities. In particular, the advent of deep genome‐resolved metagenomic sequencing has allowed for elucidation of the roles of hydrogenotrophic members of soil microorganisms in response to both atmospheric and elevated H_2_ concentrations (Xu et al., 2021). Using genome‐resolved metagenomics in microcosms spiked with a range of H_2_ concentrations, Xu et al. (2021) showed that H_2_ supplementation differentially affected bacterial phyla commonly associated with hydrogen‐uptake, by promoting the growth of hydrogenotrophic Proteobacteria and Actinobacteriota, and the abundance of genes associated with biogeochemical cycling in wetland and upland soils (Xu et al., 2021). Similarly, Wang et al. (2020) found shifts in archaeal and bacterial community composition in microcosms amended with elevated hydrogen, with specifically the ammonia‐oxidising archaeal genus *Nitrososphaera* increasing in abundance post H_2_ infusion (Wang et al., 2020). However, questions remain regarding whether the effects shown are comparable in managed agricultural soils that have distinct physicochemical properties, nutrient inputs and land‐use gradients. Moreover, agricultural practices such as the addition of fertilisers may have a significant effect on the hydrogen cycling capacity of soil‐ and plant‐associated bacteria. Thus, testing the effects of various management strategies on the capacity of different agricultural soils with distinct physicochemical and land use properties to scavenge hydrogen is paramount to understanding the downstream effects on microbe‐mediated plant growth promotion and nutritional requirements.

## A FRAMEWORK TO UNDERSTAND THE EFFECT OF AGRICULTURAL MANAGEMENT STRATEGIES ON MICROBIAL HYDROGEN CYCLING

Soil physicochemical properties can have a significant effect on the microbial community composition and ecosystem services performed by these microorganisms. In agroecosystems, soil pH, soil types, fertilisation regimes and land use types can have significant influences on the microbiome composition (Delgado‐Baquerizo et al., 2017), making it difficult to compare to studies performed in unmanaged soil ecosystems. Therefore, research should be performed on various agricultural soils with differing physicochemical properties to ascertain whether the effects of agricultural management strategies and H_2_ supplementation, in conjunction and in isolation, on hydrogen‐oxidising bacteria are generalisable across different agroecosystems. Since hydrogen‐oxidising bacteria may also act as plant‐growth promotors, further investigations should explore other benefits these bacteria can confer to plants. Similarly, the effect of hydrogen produced by hydrogen‐producing bacteria on plants, including those which produce hydrogen as a by‐product of nitrogen fixation, is an area that requires further exploration.

To determine the impacts of different agricultural management practices on the capacity of aerobic bacteria to scavenge hydrogen in agroecosystems, we propose to assess the capacity of a variety of agricultural soils subject to different management practices, such as intensive legume cropping, fertiliser addition, organic amendments, pH management strategies such as liming, and agrochemical applications. This will allow for determination of a baseline rate of hydrogen oxidation and the threshold of hydrogen oxidised by the treated soil, compared to an untreated agricultural or forest soil control. Soils amended with hydrogen concentrations above ambient could then be assessed for their ability to oxidise hydrogen, allowing for a determination of how microbial communities and biogeochemical cycling in agricultural soils respond to elevated hydrogen input. Understanding the resilience of managed agricultural soils to cope with excess hydrogen input could potentially act as a proxy for understanding how increased soil H_2_ saturation due to increasing anthropogenic emissions affects plant–soil microbiomes. Only after establishing how the agricultural microcosms respond to atmospheric and elevated concentrations of H_2_ can we answer the questions about how, for example, fertiliser addition alters the capacity of bacteria to scavenge H_2_. A systematic investigation into the hydrogen oxidation capacity of agricultural soils subject to different managements strategies will provide insights into the wide‐reaching effects of these practices on the metabolic functioning of plant–soil microbiomes, and how these may impact future agricultural productivity.

Multidisciplinary approaches combining biochemical approaches such as gas chromatography, with ‐omics based technologies such as metagenomics and metatranscriptomics, will enable a holistic understanding of H_2_ oxidation or production activity, potential microbial community structure shifts and the specific expression variations of different [NiFe] hydrogenase classes (Wang et al., 2020; Xu et al., 2021). Metatranscriptomics will enable the determination of community level functional differences, particularly Hup and Hhy, in response to agricultural management practices. In light of effects of elevated H_2_ on other functional groups, such as ammonia‐oxidising archaea (Wang et al., 2020), it would be useful to understand the response of microorganisms responsible for nitrogen cycling and other biogeochemical cycles to H_2_ fertilisation. Specifically, if an increase in the H_2_ oxidation capacity of soil bacteria results in an increase in ammonia oxidation activity by bacteria and archaea as well as production of the climate active gas nitrous oxide (N_2_O), this could cause further complications for the mitigation of nitrogen leaching and N_2_O pollution particularly in soils with high fertilisation regimes.

Similar to the methodologies employed by Maimaiti et al. (2007) and Kanno et al. (2016), it could be valuable to isolate low‐ and high‐affinity hydrogen oxidisers from the roots and rhizosphere of plants (Kanno et al., 2016; Maimaiti et al., 2007). This could expand the number of species and ecological niches capable of oxidising atmospheric and elevated concentrations of hydrogen. The H_2_ oxidation ability of these species could also be axenically assayed via gas chromatography and reverse transcriptase quantitative PCR (RT‐qPCR) in the presence of excess H_2_ and/or post addition of an agricultural management strategy (e.g., fertiliser addition) to observe any potential enzyme inhibition or stimulation effects. Hup is an especially promising target for investigation, due to the abundance of Hup within multiple lineages of the order Rhizobiales, though other uptake hydrogenases should also be looked into (Greening et al., 2016). As it is likely that hydrogen oxidation within both phyllosphere and rhizosphere bacteria is predominantly associated with mixotrophic growth, it would be interesting to explore whether bacteria possessing uptake hydrogenases use them as a mechanism to colonise plant tissue more effectively due to being able to use multiple energy sources, or whether it is restricted to epiphytic bacteria via fluorescent microscopy‐based approaches.

Despite the potential benefits of hydrogen addition to crops for yield improvement, there are still many unknowns to consider prior to commercialisation, including economic viability of adding additional H_2_ to the soils, as well as potential complications that may arise from disrupting plant‐microbe interactions by altering the proportion of H_2_ oxidising bacteria. Thus, it would be important to investigate whether these microorganisms can be manipulated to increase agricultural productivity, particularly through the enhancement of H_2_ oxidising PGPR, without having negative effects on soil microbiomes. Identification of the major microbial genera affected by H_2_ and harnessing any plant‐growth promotion properties into new biofertilisers may represent a novel biotechnological strategy applicable to a wide range of plant species. Moreover, with the use of intensive agricultural management practices, such as organic and inorganic fertilisation, use of agrochemicals and use of monoculture, significantly impact soil microbiomes, the potential for the H_2_ equilibrium to be disrupted is substantial. With the biosphere and atmosphere intertwined in part by the H_2_ cycle, changes in land management strategies will inevitably affect atmospheric composition, which in turn will affect terrestrial ecosystems. This could have wide‐reaching implications for plants, as well as planetary health, as there are currently many unknowns regarding the effects of excess versus decreased hydrogen input on plants, and how agricultural practices affect them.

## CONCLUSIONS

Understanding the ecological roles of aerobic hydrogen scavenging bacteria in agricultural soils will allow for the implementation of improved agricultural and biotechnological management strategies that can better harness the expansive benefits of hydrogen cycling in the soil environment. While there have been some indications about the positive effects of hydrogen gas on plant growth and yields, an excessive amount of hydrogen in the soil may not be adequately absorbed by hydrogenotrophs, resulting in excess climate active hydrogen to be emitted into the atmosphere. This could cause wide reaching issues for atmospheric chemistry, if for instance, fertiliser addition decreases hydrogen oxidation rates. A decrease in soil hydrogen oxidation may negatively affect both the hydrogen and other linked biogeochemical cycles, such as methane, which are reliant on hydroxyl radicals for their removal from the atmosphere. Additionally, knowledge of the effect of current agricultural management strategies on microbial diversity and hydrogen oxidation rates will have wide‐reaching implications for future soil management. Therefore, understanding the microbial dynamics necessary to maintain a balance between hydrogen oxidisers and producers in agricultural environments is of utmost importance if we are to develop new agricultural biotechnologies to help feed the world on finite land.

## AUTHOR CONTRIBUTIONS

**Zahra Fatima Islam:** Conceptualization (lead); funding acquisition (equal); investigation (lead); project administration (equal); writing – original draft (lead); writing – review and editing (lead). **Chris Greening:** Conceptualization (supporting); funding acquisition (supporting); writing – review and editing (equal). **Hangwei Hu:** Conceptualization (supporting); funding acquisition (equal); project administration (equal); supervision (lead); writing – review and editing (equal).

## FUNDING INFORMATION

This work was supported by the Australian Research Council through the Industrial Transformation Research Hub Scheme (grant number IH200100023) awarded to H‐W. H., the Australian Academy of Sciences through the Thomas Davies Research Grant for Marine, Soil and Plant Biology awarded to Z. F. I., the University of Melbourne through the Early Career Researcher Grant awarded to Z. F. I and the National Health and Medical Research Council through the EL2 Fellowship (grant number APP1178715) awarded to C. G.

## CONFLICT OF INTEREST STATEMENT

The authors declare that they have no conflicts of interest.

## Data Availability

Data sharing is not applicable as no new data were generated or analysed during this study.
